# Cases of Poisoning with Lucifer Matches

**Published:** 1853-08

**Authors:** C. Trenerry

**Affiliations:** Surgeon of the Civil Hospital, Gibraltar, and Corresponding Member of the Surgical Society of Ireland


### MATERIA MEDICA AND THERAPEUTICS.

*Cases of Poisoning with Lucifer Matches.* By C. Trenerry, Esq., Surgeon of the Civil Hospital, Gibraltar, and Corresponding Member of the Surgical Society of Ireland.—Three cases, within a very short period, wherein Lucifer matches were swallowed for the purpose of self destruction, have presented themselves to my observation. Two of them proved fatal; the third recovered.

The history of two of these cases may be related in a few words: one, a young Spanish lady, resorted to the expedient from disappointment: the other was a Spanish widow, mother of three children, who, having committed herself with a young man, aged 18, hoped by taking the poisonous substance, to induce him to marry her. The contents of the stomach were dislodged, and she recovered.

The third case was that of a native of this garrison, aged 22, who was engaged to be married, but unfortunately too close an intimacy with her lover prior to this rash act exhibited itself, she being in the seventh month of pregnancy. Her aunt states that at no time were the symptoms very urgent, nor is it known when the matches were swallowed.

The deceased first complained of pain in the stomach at 5, A. M, on Tuesday, the 17th of May, 1853, for which some "Yerba Louisa" tea was given, which relieved her. At 8, A. M., she reluctantly partook of an egg and tea for breakfast, and at eleven o'clock had some broth, but could not bear the sight of bread. During the day she complained of thirst and giddiness: in the evenicg took tea and went to bed. The next morning, the 18th, about 6, A. M., still suffering from pain and giddiness, her aunt gave her a dose of castor oil, which she ejected immediately, and frequent vomiting supervened during the day. An enema of the decoction of marsh mallows and olive oil was administered, which afforded temporary relief. In the evening, she confessed that she had taken two boxes of Lucifer matches, containing about one hundred. A

medical man was immediately consulted, who ordered an emetic and olive oil, which she said relieved her very much; but vertigo, vomiting, intense thirst, slight convulsions, and syncope, followed in rapid succession, and after midnight she became insensible, and expired at 6, A. M. on the 19th. The following day, at noon, I made a post-mortem examination, and noted the following: Body well formed, but of short stature. Face has a distorted and dirty appearance from irregular patches of congestion. Globe of the eyes very prominent; cornea of a dry and dull appearance; pupils dilated, and blood oozing from the nostrils. Livid spots about the lips, gums and tongue. *Upper part of chest and shoulders quite green and emphysematous from decomposition.* Breasts plump, and nipples marked by darkish areola and papillæ. Abdomen distended and tympanitic, *but no appearance of decomposition,* except in the fold of the groins. Vagina plugged by a dark coagulum of blood, the os uteri dilated to about the size of a shilling, and the head of a child pressed against it, as if labor had commenced prior to death. Dark feculent, or tarry-looking matter escaping from the anus. Finger nails livid, as also back part of the anus and depending parts of the sides of the body, and in some parts it is quite green. Lower extremities pale and chloritic-looking; the calf of left leg being hard and globular, as if affected with cramp. Gas escaped with a hissing noise from the apertures made by the saw in opening the skull. Dura mater dry and glistening; substance of brain so much softened as to prevent the usual examination. Ventricles very dry. Cerebellum, a perfect pulpy or custard-like mass. The encephalon throughout of a dirty cream color. Pectoral muscles soft, and easily torn. Lungs dark-colored, but crepitant; their posterior portion congested, as is usually observed after death. Pericardium contained about two ounces of dark bloody serum. Heart pale and flabby; auricles and ventricles empty. Abdominal viscera had a dull appearance. Stomach greatly distended with gas; its external surface healthy-looking. Small intestines collapsed; large intestines not so distended as is usually seen. Anterior surface of liver pale. Gall-bladder empty. Spleen very dark-colored. Uterus contained a well-formed but dead female fœtus, of about seven or eight months, floating in the liquor amnii, the membranes being unbroken. The cellular tissue and depending or posterior portions of the liver, pancreas, spleen, and kidneys, unusually congested, amounting almost to extravasation of blood. The pharyngeal and cardiac end of the œsophagus of a dark or bruised color; the canal appears contracted and the mucous membrane dry, one rounded carbonacious head of a match adhering to its surface. Stomach contained about four ounces of a dark, unctuous treackly-looking fluid. The mucous membrane of a whitey-brown color, and the cardiac or larger end thickly vesicated, as if scalded; the middle portion retained its rugous appearance, although much thicker and paler than usual. The pylorous quite smooth, pale, and thickened, as also the mucous membrane of duodenum, which, with about three feet of the jejunum, contained a similar black-looking fluid as the stomach. The remaining portion of small intestines were merely lined with a creamy feculent substance interspersed with patches of greenish or bilious matter.—*Dublin Medical Press.*

